# Giant Dissecting Aneurysm of the Internal Carotid Artery in a 35-Year-Old Patient: A Case Report

**DOI:** 10.7759/cureus.67353

**Published:** 2024-08-20

**Authors:** Amira Kamel, Ligia G Tataranu, Gheorghe Vasile Ciubotaru, Adriana Solomon, Radu Eugen Rizea

**Affiliations:** 1 Neurosurgery, Emergency Clinical Hospital Bagdasar-Arseni, Bucharest, ROU; 2 Neurosurgery, Carol Davila University of Medicine and Pharmacy, Bucharest, ROU

**Keywords:** neurovascular, neurosurgery, endovascular embolization, dissecting aneurysm, internal carotid artery

## Abstract

Giant dissecting aneurysms of the internal carotid artery are extremely uncommon, particularly in young adults. In this report, we provide a case of a 35-year-old male patient who experienced severe headaches, double vision, paralysis of the left abducens nerve, trigeminal neuralgia, nausea, and vomiting. The cerebral MRI showed an intensely gadolinophilic lesion following the left internal carotid artery route from the petrous canal; it also caused an internal deviation of the cavernous route of the internal carotid artery with a fluid heterogeneous area that pushed the cavernous dura mater (including the Gasser ganglion) on the free cisternal route of the trigeminal nerve. Furthermore, the cerebral angiography revealed a giant dissecting aneurysm at the C2-C4 junction of ICA, anteriorly oriented with perilesional stenoses. The interdisciplinary medical team determined that the most optimal therapeutic strategy would involve coil embolization, and the giant left ICA aneurysm was occluded along with the left ICA, with 15 giant platinum coils. Following the successful intervention, the patient experienced a remarkable clinical outcome, characterized by an immediate reversal of the majority of the symptoms. Although we were not certain of the alleviation of symptoms after the endovascular treatment, fortunately, the results were beyond expectations.

## Introduction

Giant dissecting aneurysms of the internal carotid artery (ICA) are very rare, and the average reported incidence is approximately 3 per 100,000, with no specific etiology in the majority of the reported cases [1]. However, some etiologic factors have been reported in up to 41% of the patients, like minor trauma, sports or physical activity, headbanging, extended periods of head flexion, as a complication of general anesthesia, or even family history [2], and only 5% of the patients are asymptomatic [2].

When it comes to this type of vascular lesion, several diagnostic options are available and many of them were also approached in our case. A cerebral CT scan is usually the first option as the primary suspected origin of symptoms caused by mass effect is a tumor. Furthermore, a CT angiography could provide important details about the arteries, as well as the blood flow. Magnetic resonance imaging is usually the second option if no conclusive result is provided by the CT scan. Magnetic resonance angiography provides much more detail regarding the size, type, or anatomical localization of the aneurysm. However, due to the amount of vascular details provided, cerebral angiography remains the gold standard in diagnosing cerebral aneurysms. The choice of therapeutic strategy is contingent upon criteria such as the underlying cause, the patient's age, vascular anatomy, collateralization possibility, and any coexisting medical conditions, and remains a subject of ongoing debate. However, it can involve both a medical and neurosurgical approach, as well as endovascular aneurysm repair [1]. This article presents a case of a giant dissecting aneurysm involving C2-C4 segments of the ICA. The aneurysm exhibited typical symptoms of a cavernous aneurysm and there was no previous medical history indicating any recent causes.

## Case presentation

A 35-year-old male patient was admitted to our neurosurgical department in the Clinical Emergency Hospital Bagdasar-Arseni, Bucharest, for refractory agonizing headaches, diplopia, left abducens nerve palsy, ophthalmic branch trigeminal nerve (V1) neuralgia, sickness, and vomiting. The symptoms manifested two weeks before hospitalization and progressively increased in severity. The patient's medical history was unremarkable except for a childhood appendectomy and a documented allergy to penicillin.

**Figure 1:**
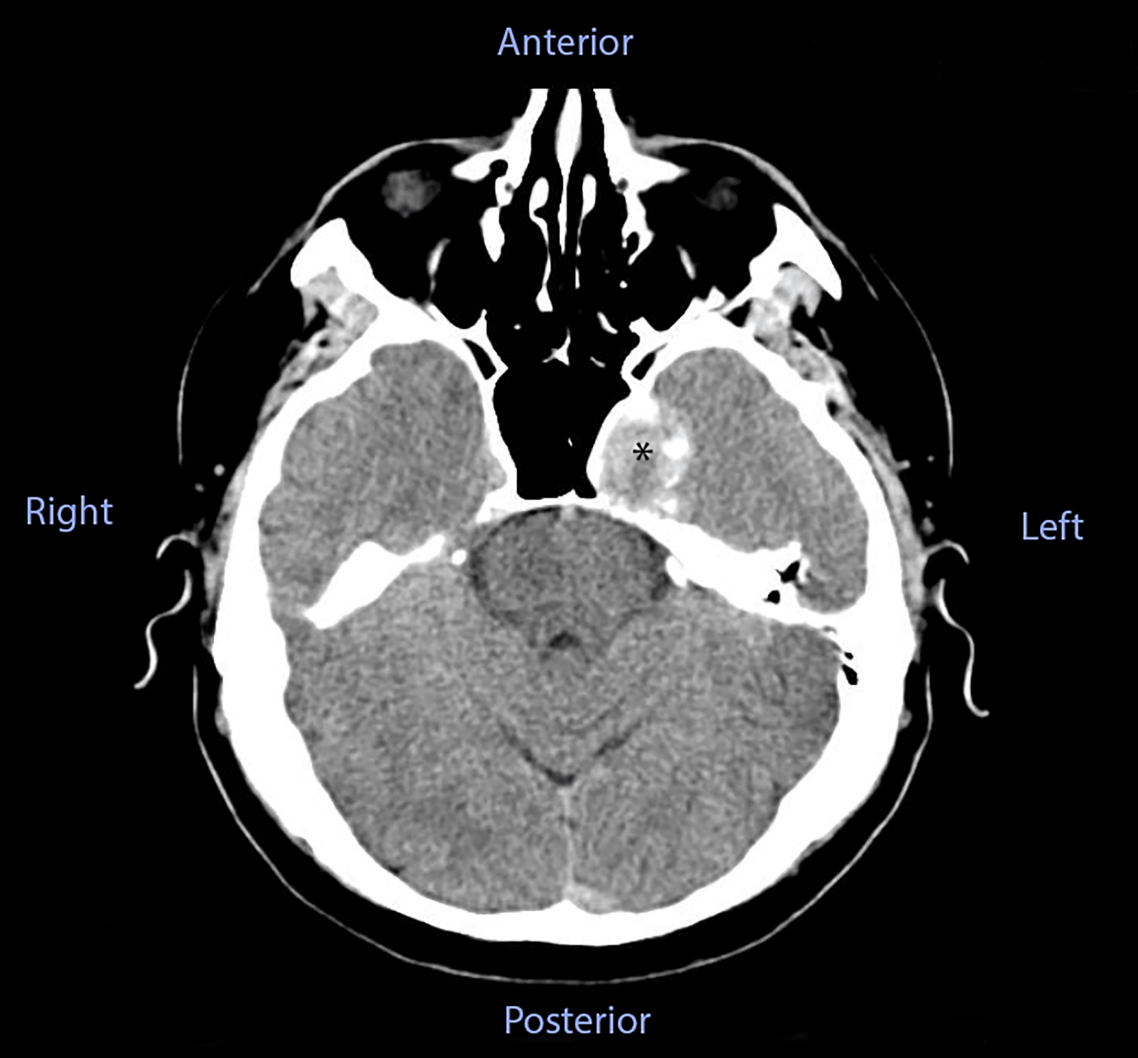
Cerebral CT scan of our patient before being admitted to our hospital. The asterisk marks the discovered lesion on the left side.

The patient had a cerebral CT scan in another medical service one week before coming to our department, which revealed a nodular, native, hyperdense lesion with peripheral calcifications, and its length and breadth were 28 mm and 27 mm, respectively. The lesion was extra-axially developed, starting from the left greater sphenoid wing, with a mass effect over the left sphenoid sinus, causing cortical bone ballooning expansion and thinning (Figure 1).

**Figure 2:**
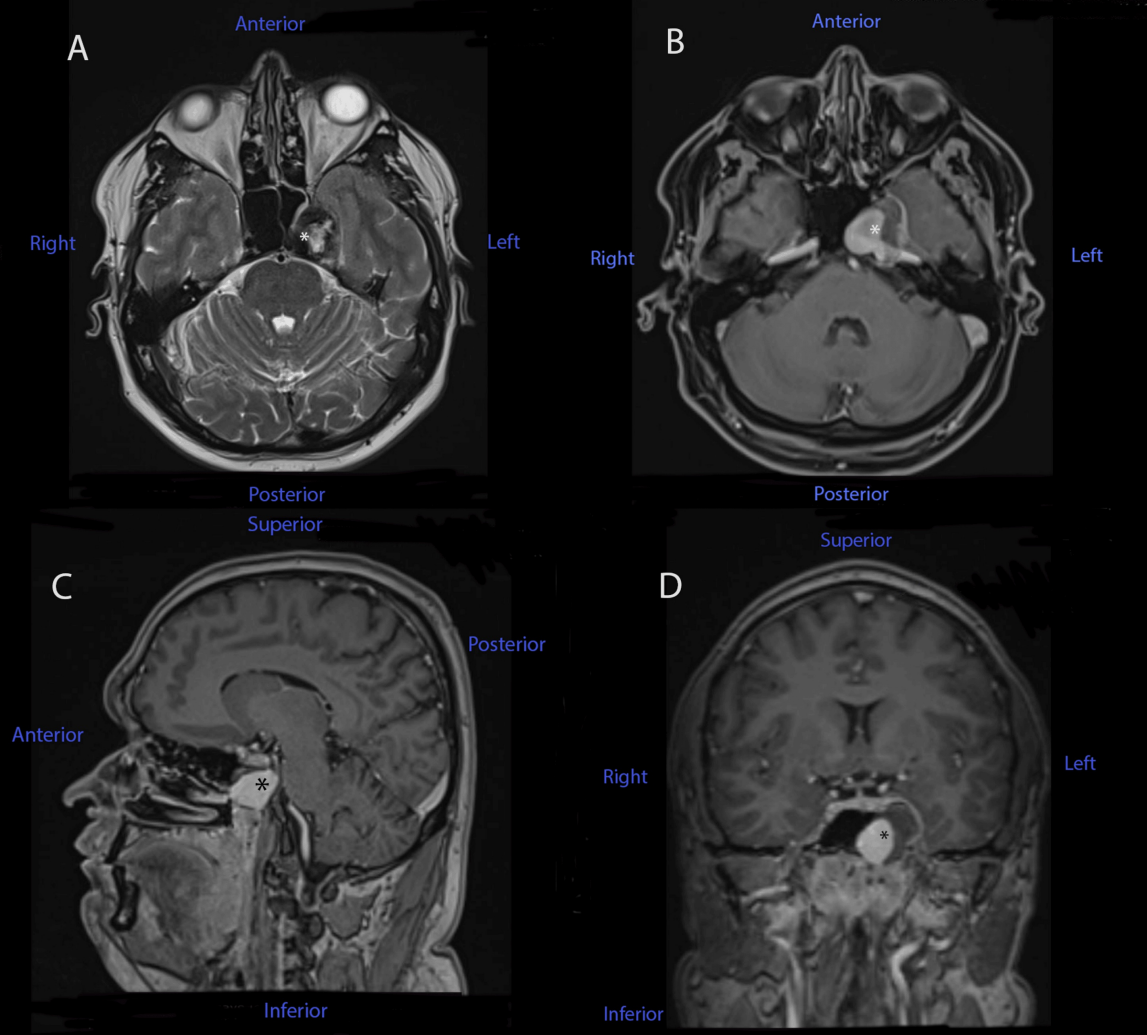
MRI brain images of our patient, before being admitted to our hospital. (A) T2-weighted axial sequence; (B) T1-weighted contrast-enhanced axial image; (C) T1-weighted contrast-enhanced sagittal image; (D) T1-weighted contrast-enhanced coronal sequence. Note that the asterisk marks the presence of the lesion in each sequence.

After receiving this result, the patient was advised to undergo a contrast-enhanced cerebral MRI, which he did after two days. The brain MRI revealed a lesional area that affected both the left petrous apex and the left cavernous area, with an invasion of the left sphenoidal cavity (Figure 2). The lesion was intensely gadolinophilic and followed the left internal carotid artery route from the petrous canal; it then enlarged to the apex where it caused an internal deviation of the cavernous route of the internal carotid artery with a fluid heterogenous area that pushed the cavernous dura matter (including the Gasser ganglion) on the free cisternal route of the trigeminal nerve. The described lesion was consistent with a vascular lesion, possibly a cavernous carotid aneurysm (length: 26m m; breadth: 19 mm). Five days later, the patient presented at our hospital.

**Figure 3:**
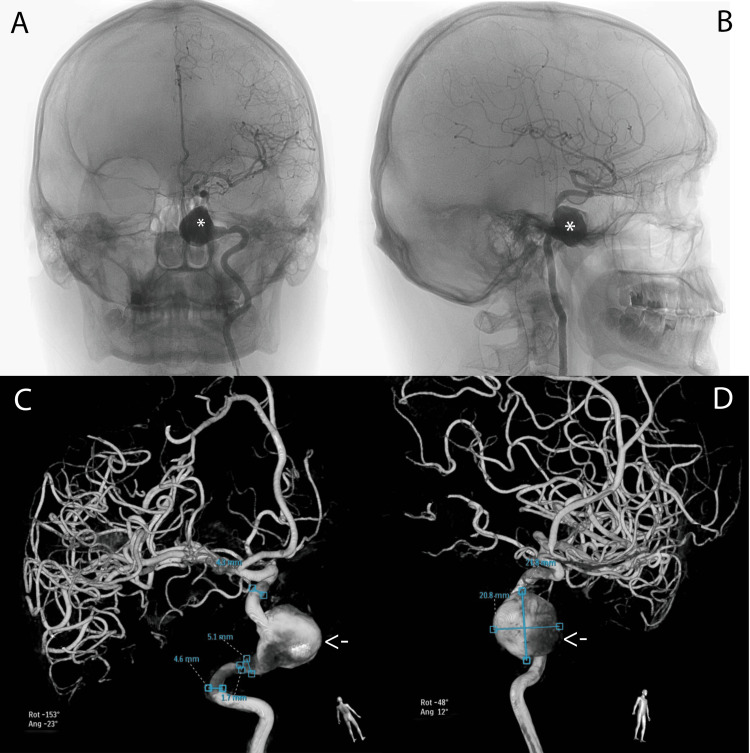
Cerebral angiography of our patient at admission to our department. (A) Coronal view; (B) Sagittal view; (C and D) 3D renderings with measurements and positioning scales. The asterisks and arrows mark the presence of the lesion on every image.

**Figure 4:**
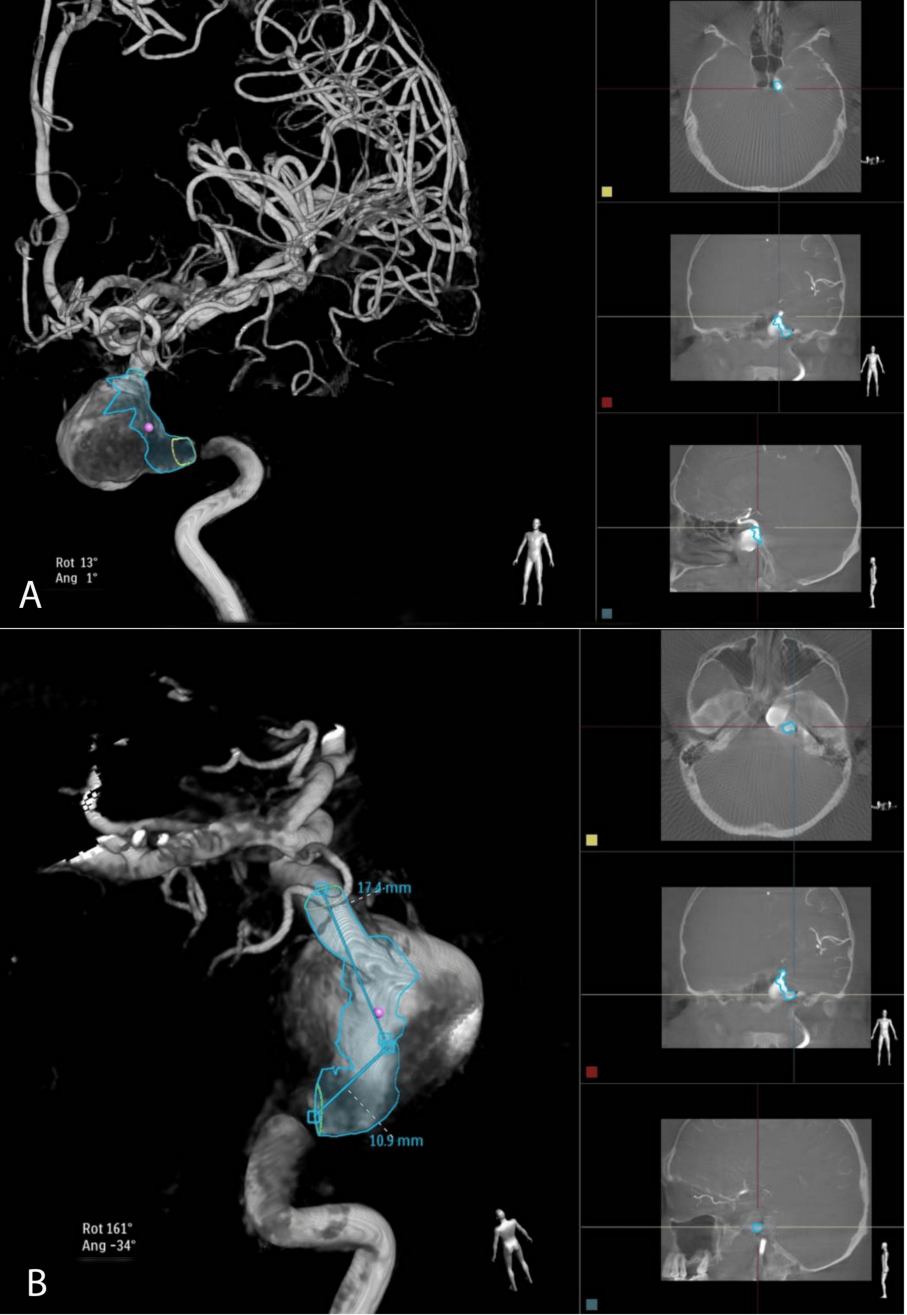
Supplementary 3D renderings of the cerebral angiography of our patient at admission to our department, with measurements and positioning scales (A and B).

After being admitted to our department, a cerebral angiography was performed (Figures 3-4). The results described a giant dissecting aneurysm inserted at the C2-C4 junction of ICA, anteriorly oriented, with a maximal diameter of 2.5 cm (approximately 2.2 cm) and a neck of 17 mm. The images also showed perilesional stenoses with diameters of 1.7 mm and 3 mm.

After a thorough analysis of the case, the multidisciplinary team, which comprised neurosurgeons, interventional neuroradiologists, and neurologists, concluded that the Willis circle is complete and the possibility of collateralization exists not only from the right ICA via the anterior communicating artery, but also from posterior circulation via the posterior communicating artery. Therefore, in case of left ICA obstruction, vascularization would still be provided. Thus, the best therapeutic approach considered would be endovascular embolization with coils and the endovascular coiling was planned.

The procedure was performed under local anesthesia (lidocaine 1%, 10 ml) at the right inguinal fold. Subsequently, a guiding catheter was used (Neuronmax 6F), which served to navigate until the petrous segment (C2) of the left ICA, and then 5000 IU of heparin was administered. The giant aneurysm was detected with a maximum diameter of approximately 32 mm at the lacerum segment (C3) of the left ICA, with a vascular narrowing (75%) not only before, but also after the aneurysm. The balloon occlusion test was done on the left ICA (5.5/30 balloon with a BMW 0.014 microguide), and after 20 minutes of occlusion with a maximal systolic arterial tension of 140 mmHg, the interventional neuroradiologist considered that the internal carotid artery could be sacrificed along with the aneurysmal sac. The right ICA and vertebral artery were also verified.

**Figure 5:**
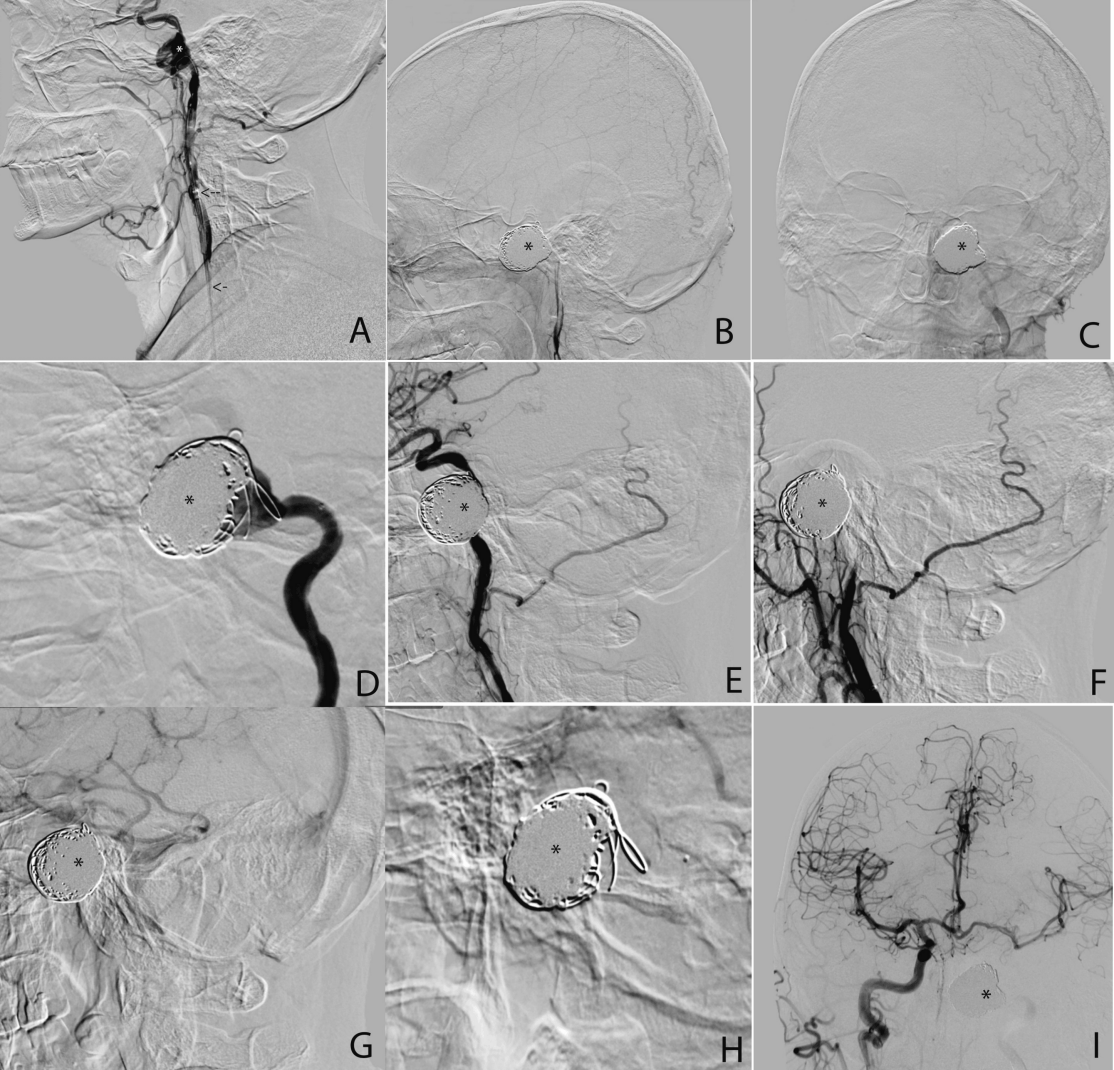
Intra-interventional and post-interventional images A: Intra-interventional registration images (sagittal view); the black arrows show the Neuronmax 6F guiding catheter, while the white asterisk marks the intracranial aneurysm before embolization. B and C: Post-interventional images, sagittal (B) and coronal views (C); the black asterisk shows the intracranial aneurysm, now embolized with platinum coils. D, E, F, G, and H: Details from the sagittal images of the embolized aneurysm of our patient, with the black asterisks marking the coils from the now embolized aneurysm. I: Post-interventional image, showing left and right cerebral hemisphere vascularization, with the black asterisk marking the embolized aneurysm.

The giant left ICA aneurysm was occluded along with the left ICA, with 15 giant platinum coils. The results were successful (Figure 5), especially since the patient had no further postinterventional neurological deficits. The left cerebral hemisphere vascularization is now routed from the right ICA via the anterior communicating artery, but also from the posterior circulation via the left posterior communicating artery. The entire procedure was successfully performed without any incidents.

## Discussion

The medical literature reports many cases of dissecting aneurysms of the head and neck, which are described as vascular diseases that imply the presence of a hematoma in the wall of the artery; however, dissecting aneurysms of the ICA are rare entities [1]. The etiology of this vascular pathology is either traumatic or spontaneous, and although the mechanisms are very different, in reality, dissection occurs as a combination of extrinsic forces and intrinsic weakness [3]. It has been demonstrated that in spontaneous dissections, patients are significantly older, often with cardiovascular pathologies and hypercholesterolemia [3].

The rarity of our case is a given the typical patient profile of this condition. Our patient was young, a 35-year-old male, with no medical history other than an appendectomy in childhood and a penicillin allergy. No history of recent or former head or neck trauma was reported in our case.

It has been stated that in patients with spontaneous dissection of the carotid arteries, abnormal elastic properties were discovered, as higher stiffness of the arterial wall was combined with circumferential wall stress, and these factors increased the risk of dissections [4]. Furthermore, the aneurysmal growth may be due to recurrent hemorrhage into the wall or slow expansion of the true aneurysmal wall [5]. ICA aneurysms of the cavernous area usually present with cavernous sinus syndrome because of the mass effect [5], which was also the case with our patient, who complained of agonizing headaches that were refractory to the usual treatment, diplopia, left abducens nerve palsy, trigeminal neuralgia, as well as sickness and vomiting, with symptoms worsening over time. Besides the mass effect in areas in which pain fibers are present, the exact mechanism of headache in unruptured aneurysms has not yet been elucidated. However, some theories have been proposed. One of the theories suggests that the hemodynamic stress on the aneurysmal wall could be a source of pain if there are arteries in which pain fibers are present. Another theory suggests that the irritation of the adjacent dura mater could be the main cause responsible for this phenomenon. A distinct theory refers to micro-leaks of the aneurysm which can mimic a subarachnoid hemorrhage, causing headaches. If we consider these theories, the results could be compatible with symptom improvement in our case. Although we were not certain of the alleviation of headaches after the endovascular treatment, fortunately, the results were beyond our expectations.

Typical neuroimaging findings were described in this case. The initial assessment was a cerebral CT scan, which revealed a nodular, native, hyperdense lesion with peripheral calcifications, apparently extra-axially developed, starting from the left greater sphenoid wing, with a mass effect over the left sphenoid sinus, causing cortical bone ballooning expansion and thinning. Subsequently, after a medical recommendation, the patient had a contrast-enhanced brain MRI, which revealed a lesional area that affected both the left petrous apex and the left cavernous area, with an invasion of the left sphenoidal cavity. The lesion was intensely gadolinophilic and was consistent with a vascular lesion, raising the possibility of a cavernous carotid aneurysm. Finally, after being admitted to our hospital, we performed a cerebral angiography, which diagnosed a giant dissecting aneurysm at the C2-C4 segments of the ICA, anteriorly oriented, with perilesional stenoses. These typical findings were previously described in the medical literature, although cases of young patients with a non-traumatic history and no other comorbidities were very rare [6-9].

When it comes to the therapeutic management of ICA aneurysms, multiple factors should be considered, such as age, comorbidities, type, and size of the aneurysm, as well as the balance between the risks and benefits of every therapeutic option. Given the fact that every patient is different, a multidisciplinary approach is the best option when choosing to manage this pathology. Thus, neurosurgeons, neuroradiologists, and neurologists are the main experts of this multidisciplinary team. In our case, this team considered that the best therapeutic management of the giant dissecting ICA aneurysm would be endovascular embolization with coils. Several elements were taken into account in making this decision, such as the morphology of the aneurysm and the risk of vessel injury that could potentially lead to hemorrhagic/ischemic complications. Currently, endovascular embolization with coils can provide near-complete aneurysmal occlusion in more than 90% of the cases, with less than 1% neurological complications [6], and the main complication of this method is represented by coil migration during embolization [10]. Fortunately, excellent results were also obtained in our case. It is worth mentioning that a flow-diverting stent would have been another viable option. This therapeutic method deflects the blood flow from the aneurysm, thus promoting natural thrombosis. However, this technique was not available to our team at the time.

This case is rare given the age and the sex of the patient. The majority of patients with this pathology are women, the median age is above 50 years and they also have other comorbidities. Young patients presenting these types of lesions usually have a traumatic history regarding the head or neck, or they have associated genetic or chronic illnesses [1, 9, 11]. Thus, a lack of predisposing factors confers uniqueness to our case while providing more insight into this pathology. Further research is needed in order to determine the exact etiology of these cases, as well as to provide the best therapeutic approach, as the management of this entity is still controversial [6, 12].

## Conclusions

When it comes to rare cases of cerebrovascular entities, such as a giant dissecting ICA aneurysm in a young, healthy patient, many factors should be taken into account. Since there is no protocol for such pathology, a multidisciplinary team must adapt to provide the best therapeutic management while considering the balance between risks and benefits. 

Although the exact correlation between headaches and unruptured aneurysms had not yet been elucidated, we concluded that the hemodynamic stress on the aneurysmal wall could be a potential source of pain or irritation to the adjacent dura mater, as well as micro-leaks of the aneurysm which can mimic a subarachnoid hemorrhage. Further research is needed to gain more insight and provide a better understanding of such cases, and we are hopeful that this particular report will make even a small contribution to this goal.
